# Chronobiology of fracture repair: local molecular clocks, rhythm protection, and time-anchored translation

**DOI:** 10.3389/fendo.2026.1882669

**Published:** 2026-09-07

**Authors:** Wenjie Lu, Nana Jiang, Jiaming Zhang, Jinzhong Wang, Zhimin Wu, Jianmiao Jin

**Affiliations:** 1 Shaoxing TCM Hospital Affiliated to Zhejiang Chinese Medical University, Shaoxing, China; 2 Yangming College, Shaoxing Polytechnic Institute, Shaoxing, China; 3 Huai’an Traditional Chinese Medicine Hospital, Huai’an, China

**Keywords:** bone regeneration, circadian rhythm, fracture healing, molecular clock, perioperative care, rhythm protection

## Abstract

Fracture repair is a prototypic regenerative process in adult tissues and proceeds through a temporally organised sequence of haematoma formation and inflammation, cartilaginous callus formation, hard callus mineralisation, and callus remodelling. Conventional fracture research has primarily attributed variation in healing outcomes to mechanical stability, vascular supply, inflammation, metabolic bone status, drug exposure, nutrition, and rehabilitation loading. Time, by contrast, has often been treated as a background variable. Emerging evidence from circadian biology and molecular-clock research indicates that bone turnover markers, endocrine signals, immune responses, vascular function, sleep-wake organisation, and the activities of osteoblasts, osteoclasts, chondrocytes, macrophages, and endothelial cells are subject to rhythmic regulation. In particular, Per2::Luc oscillation at the mouse femur fracture site and its responsiveness to parathyroid hormone (PTH) provide direct preclinical evidence that a local molecular clock can operate within the fracture repair niche. Fracture chronobiology, however, remains at an early translational stage. Current evidence is derived largely from animal models, cell studies, human bone-marker physiology, sleep and circadian-disruption experiments, and perioperative literature. Direct time-resolved clinical data in human fracture repair remain sparse. Accordingly, the available evidence does not justify recommending a specific clock time for surgery, drug administration, blood sampling, loading, or rehabilitation to improve fracture healing. We argue that the immediate translational value of fracture chronobiology lies not in premature clinical chronotherapy but in moving fracture research and perioperative care from a time-agnostic to a time-anchored framework. The perioperative period should be regarded as a rhythm-vulnerable window in which avoidable disruption from trauma, pain, anaesthesia, nocturnal light, sleep fragmentation, and medication exposure may be reduced. At the same time, fracture studies should systematically record the timing of injury, admission, anaesthesia, surgery, drug administration, biological sampling, sleep, light exposure, activity, rehabilitation, and imaging follow-up. A time-anchored research framework can clarify whether rhythmic variables influence callus formation, mineralisation, remodelling, nonunion, and functional recovery, thereby providing a measurable foundation for future fracture chronobiology trials.

## Introduction

Fracture repair is one of the best-characterised regenerative processes in adult tissues, yet its clinical outcomes remain heterogeneous. Most fractures heal uneventfully after appropriate reduction, stable fixation, and rehabilitation. Delayed union, nonunion, poor callus quality, and incomplete functional recovery remain major problems in orthopaedic trauma, particularly among older adults and patients with osteoporosis, diabetes, smoking exposure, severe soft-tissue injury, or multimorbidity. Classic explanatory frameworks have focused on local mechanical stability, vascularity, inflammation, metabolic bone status, medication exposure, nutrition, and rehabilitation loading. One dimension, however, has received less systematic attention: fracture repair occurs within a highly time-organised biological system (1–6).

Fracture repair is not a homogeneous or static event. It is a staged regenerative sequence in which haematoma formation and inflammation, mesenchymal progenitor recruitment, cartilaginous callus formation, endochondral ossification, hard callus mineralisation, and callus remodelling overlap in time. Each phase is shaped by distinct dominant cell populations, signalling pathways, and mechanical contexts. Early repair is driven by inflammatory-cell recruitment, cytokine release, hypoxia, and vascular responses; intermediate repair depends on chondrogenic differentiation, cartilaginous callus formation, and vascular invasion; later repair requires coupled osteoblast and osteoclast activity to complete mineralisation and remodelling. Thus, the same molecular signal or clinical intervention may have different effects depending on the repair stage. Stimuli delivered too early, too late, or for too long may alter repair quality.

Circadian biology offers an endocrine–temporal perspective for interpreting this complexity. Circadian rhythms are endogenous approximately 24-h processes coordinated by central and peripheral clocks, whereas clock time, sleep, meals, activity, and light are external or behavioural time cues rather than direct measures of internal phase. The molecular clock is organised around transcription–translation feedback loops involving CLOCK/BMAL1, PER/CRY, and REV-ERB/ROR, which coordinate endocrine signalling, metabolism, inflammation, cell proliferation, and repair (7–10). Bone is not a passive target of this system: bone and cartilage homeostasis, osteoporosis, and musculoskeletal metabolism are influenced by circadian mechanisms (11–13). Fracture repair may therefore be shaped by systemic endocrine rhythms and local repair-clock activity, but external clock time should not be treated as a surrogate for biological phase.

A critical distinction is needed. A chronobiological basis for fracture repair does not imply that mature clinical chronotherapy already exists. Recent reviews have mainly addressed skeletal homeostasis, osteoporosis, cartilage biology, or general musculoskeletal rhythms; they have not integrated an injury-induced fracture niche with repair-stage dependence, perioperative rhythm vulnerability, and a practical reporting agenda. In addition, many fracture studies do not record injury, surgery, dosing, sampling, sleep, light, loading, or imaging time. Temporal effects may therefore be obscured or misclassified.

**Figure 1:**
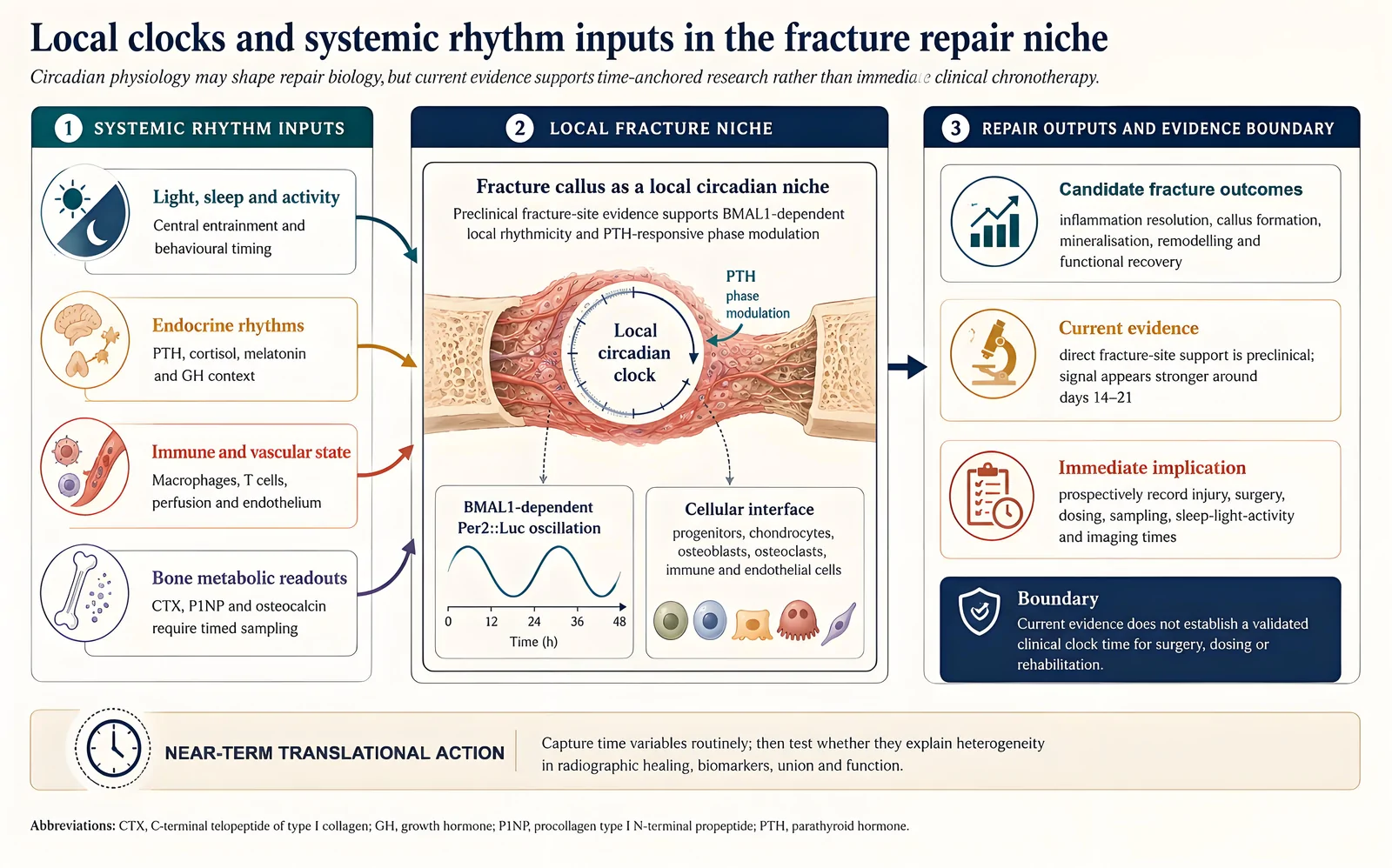
Evidence-bounded model of local molecular clocks and systemic rhythm inputs in the fracture repair niche. BMAL1-dependent Per2::Luc oscillation and PTH-responsive phase modulation are direct preclinical fracture-site observations. Links from systemic inputs or local clock activity to callus formation, mineralisation, union, and function are candidate relationships for prospective testing, not validated healing effects or clinical treatment times.

The central premise of this review is that the immediate value of fracture chronobiology lies in an endocrine–circadian, time-anchored framework rather than premature prescription of treatment hours. We synthesise fracture-site clock evidence, stage-specific repair biology, and perioperative rhythm disruption; distinguish direct fracture evidence from adjacent skeletal and physiological evidence; and propose feasible reporting and research priorities. This fracture-specific synthesis links local biology to perioperative care while preserving a clear boundary between mechanistic plausibility and clinical recommendation. The proposed evidence-bounded model is shown in **Figure 1**.

## Search strategy and evidence classification

This narrative review was prepared with reference to the Scale for the Assessment of Narrative Review Articles (SANRA), particularly its emphasis on clinical relevance, explicit aims, transparent literature searching, appropriate referencing, scientific reasoning, and adequate presentation of outcome-relevant data (14). This article is not a systematic review and does not include formal risk-of-bias scoring or meta-analysis. Nevertheless, we used a structured narrative search and prespecified evidence classification to improve transparency.

PubMed/MEDLINE, Embase, Web of Science, Scopus, and the Cochrane Library were searched primarily from January 2000 to January 2026, with older foundational articles included where relevant; a targeted PubMed update was completed on 6 August 2026 for the revision. Search concepts combined fracture healing or bone regeneration with circadian rhythm, molecular clock, clock genes, bone turnover markers, PTH or teriparatide, melatonin, sleep and perioperative disruption, surgical timing, antiresorptives, NSAIDs, vitamin D, nutrition, exercise, and mechanical loading. Eligible evidence included basic and animal studies, human physiological studies, trials, observational studies, systematic reviews, meta-analyses, and relevant methodological or consensus articles. We excluded records unrelated to fracture repair, skeletal biology, chronobiology, perioperative care, or time-dependent therapeutics; conference abstracts without sufficient data; unverifiable reports; and duplicate publications.

Evidence was organised into domains defined by proximity to fracture repair rather than ranked as a hierarchy of methodological quality. The domains comprised direct fracture-site or fracture-model chronobiology; rhythmic mechanisms in repair-relevant skeletal, immune, and vascular cells; human bone-turnover and endocrine physiology; perioperative and care-process evidence; and indirect evidence from osteoporosis, mechanical loading, nutrition, sleep, or general chronotherapy. Direct fracture evidence anchored the principal claims, whereas adjacent mechanistic and physiological evidence was used to generate explicitly bounded hypotheses. The evidence domains are summarised in **Table 1**.

**Table 1:** Evidence domains, representative studies, and translational relevance in fracture chronobiology.

Evidence domain	Representative evidence	What it supports	Main limitation	Evidence source and fracture specificity
Direct fracture-site molecular clock evidence	Ex vivo mouse femur fracture Per2::Luc oscillation and PTH responsiveness (15)	A measurable BMAL1-dependent, stage-related local clock signal can operate in the fracture niche	Single ex vivo reporter model; no human fracture-outcome validation	Direct fracture-site preclinical evidence
Human bone-turnover rhythmicity	Diurnal CTX, osteocalcin, and P1NP studies; constant-routine CTX study (16–19)	Sampling time is biologically meaningful	Not fracture-specific and influenced by feeding, sleep, posture, and activity	Established human physiological evidence
Sleep and circadian disruption	Controlled sleep-restriction/circadian-disruption studies (20, 21)	Bone metabolism responds to sleep and circadian state	Small non-fracture studies without callus or union endpoints	Human physiological; hypothesis-generating for fracture
Repair-relevant cellular clocks	Osteoblast, osteoclast, BMSC, chondrocyte, immune, and vascular studies (22–41)	Cells central to repair have temporal regulatory capacity	Mostly homeostasis or adjacent-tissue evidence	Indirect mechanistic evidence
Perioperative rhythm disturbance	Anaesthesia, postoperative sleep, and melatonin/ramelteon literature (42–49)	The perioperative period can disrupt sleep–wake and endocrine–immune organisation	Fracture healing is rarely an endpoint	Clinically relevant supportive evidence
Clinical timing of orthopaedic care	Hip-fracture surgical-delay studies and meta-analyses (50–53)	Care-process timing affects clinical outcomes	Delay is not circadian phase; confounding is substantial	Human clinical evidence for process time, not chronotherapy
Bone-active therapy timing	Antiresorptive initiation, fracture PTH trials, NSAID timing, and vitamin D studies (54–64)	Initiation, dose, duration, and phase are separable exposures	Only NSAID timing has direct fracture-model outcome evidence; PTH clock-time data are non-fracture	Mixed human fracture and preclinical evidence
Loading, feeding, and vulnerable states	Intact-bone loading, postmenopausal exercise/feeding, diabetes, ageing, and osteoporosis studies (63–77)	Defines testable modifiers and priority cohorts	Predominantly indirect; safety and fixation constraints limit translation	Hypothesis-generating for fracture-specific studies

BMSC, bone marrow mesenchymal cell; BTM, bone turnover marker; CTX, C-terminal telopeptide of type I collagen; P1NP, procollagen type I N-terminal propeptide; PTH, parathyroid hormone.

## Chronobiological basis of fracture repair

### Human bone-turnover rhythms

Daily variation in human bone metabolism provides one of the most robust indirect evidence layers for fracture chronobiology. Early studies showed that serum C-terminal telopeptide of type I collagen (CTX) fluctuates substantially over 24 h, typically increasing at night or in the early morning and decreasing during daytime, with additional modulation by feeding, sleep, activity, and posture. Osteocalcin also shows diurnal variation. Procollagen type I N-terminal propeptide (P1NP) tends to show weaker day-night variation, although rhythmicity can be detected in selected populations and controlled settings. A recent constant-routine protocol, controlling for sleep, food intake, posture, and light exposure, confirmed a robust intrinsic circadian rhythm in serum CTX, whereas evidence for P1NP rhythmicity was less pronounced (16–19). Bone resorption rhythms therefore appear more consistent than bone formation rhythms.

This evidence has immediate methodological implications for fracture studies. If the sampling times of CTX, P1NP, osteocalcin, cortisol, melatonin, and inflammatory mediators are not recorded or standardised, biomarker results may be confounded by diurnal variation. Morning, afternoon, and night samples should not be pooled without consideration of collection time. When fixed sampling is impractical, the sampling time should at least be recorded and adjusted for, or tested in sensitivity analyses. Even in studies that do not test a rhythm intervention, time should be treated as part of the basic measurement structure.

Sleep and circadian disruption also affect bone turnover markers. Small human experimental studies have shown that sleep restriction combined with circadian disruption can reduce bone formation markers over several weeks, with some evidence of altered bone resorption markers (20, 21). These studies were not conducted in fracture patients and did not assess callus formation or union. They do, however, show that bone metabolism is measurably responsive to sleep and circadian state. In fracture patients, perioperative sleep fragmentation, nocturnal light exposure, pain, anxiety, analgesics, and sedatives may collectively alter the systemic background in which repair occurs. These variables are therefore worth recording and analysing.

### Clocks in repair-relevant cells

Repair-relevant cells have circadian regulatory capacity, but most evidence is not fracture-specific. Osteoblast and osteoclast clocks influence matrix deposition, resorption, and cell coupling; bone marrow mesenchymal cells can display synchronised clock-gene oscillation; and chondrocyte clocks regulate cartilage matrix homeostasis (22–28). These findings provide a mechanistic substrate for temporal sensitivity across the callus, but they do not establish rhythmic function in each cell population during fracture healing.

Chondrocyte evidence is particularly relevant to callus-mediated secondary healing because PTH can reset oscillation in cultured murine femoral cartilage (28). However, permanent articular cartilage, the developmental growth plate, and transient injury-induced callus are biologically distinct. These models therefore support a fracture-specific question rather than a direct clinical inference.

### Local fracture-site clocks

The strongest direct evidence comes from Kunimoto et al., who used Per2::Luc reporter mice and real-time bioluminescence imaging of ex vivo fractured femora under external fixation. The fracture region showed an approximately 24-h oscillation; in Per2::Luc;Bmal1-deficient mice, signal persisted but rhythmicity was lost, indicating BMAL1 dependence (15).

The signal was stage-related, appearing stronger around postoperative days 14–21 and weakening during later remodelling. This pattern is compatible with dynamic coupling to callus transition, but reporter intensity is not a surrogate for callus quality, union, or mechanical strength.

PTH shifted the peak timing of Per2::Luc activity at the fracture site and growth plate while preserving an approximately 24-h rhythm (15). This demonstrates endocrine responsiveness of a local clock signal, not that morning or evening PTH dosing improves fracture healing.

Thus, direct fracture-site chronobiology currently rests heavily on a single ex vivo reporter model. It establishes that a measurable, BMAL1-dependent, PTH-responsive rhythm can operate in the mouse fracture niche, but clinical operability requires independent replication and prospective human validation.

## Stage-specific chronobiology of secondary fracture healing

**Figure 2:**
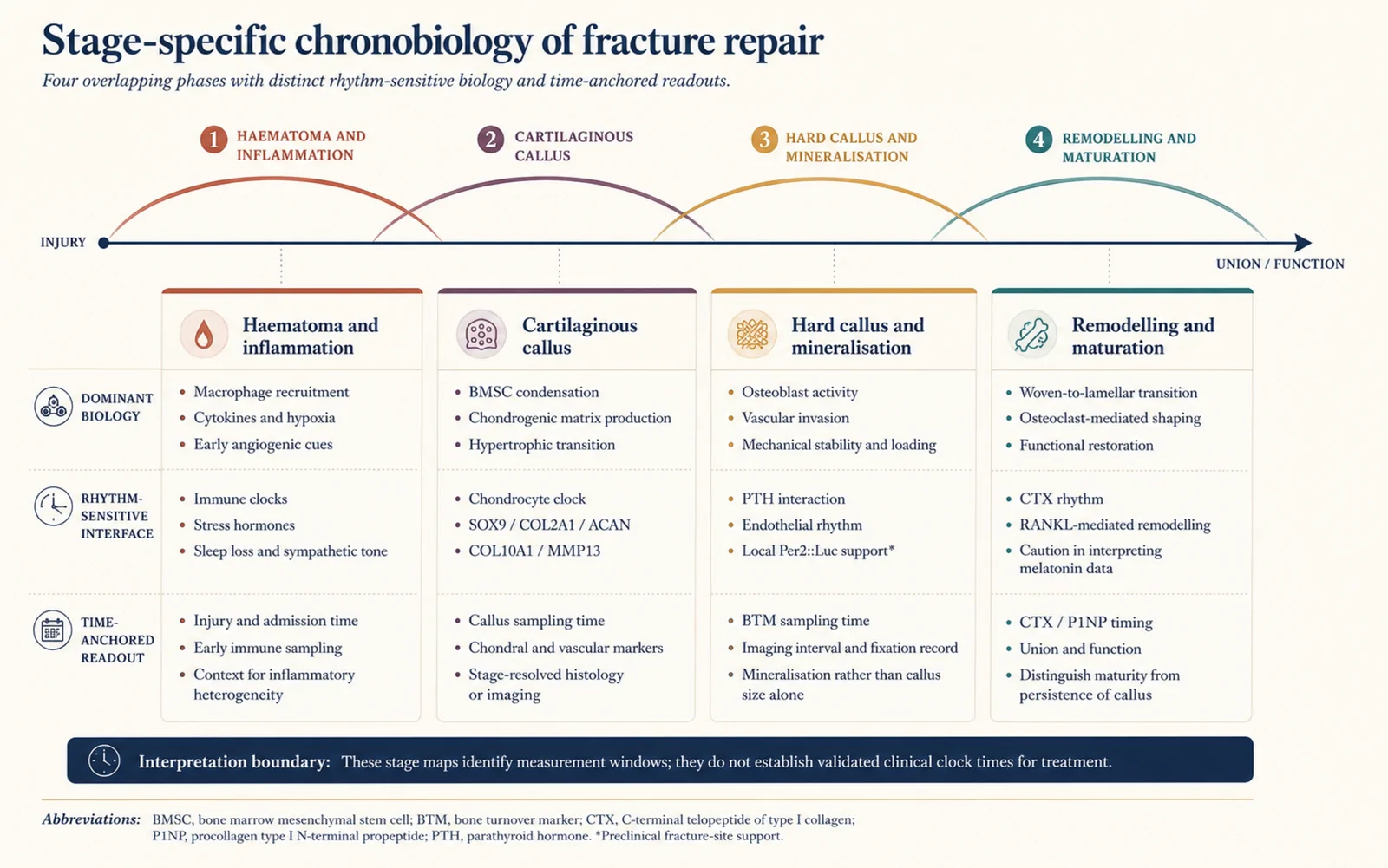
Stage-specific chronobiology of callus-mediated secondary fracture healing. The four overlapping phases are mapped to dominant biology, rhythm-sensitive interfaces, and time-anchored readouts. The asterisk beside “local Per2::Luc support” denotes preclinical fracture-site rhythmicity observed around postoperative days 14–21; it does not indicate a demonstrated benefit in hard-callus formation. The framework identifies measurement windows and does not establish treatment times.

The value of fracture chronobiology is not in broadly asserting that rhythms affect bone healing, but in identifying repair-stage-specific temporal dependencies. The framework below primarily describes callus-mediated secondary fracture healing; its applicability to primary cortical healing or predominantly intramembranous repair requires separate evaluation. Inflammation, cartilaginous callus, hard callus, and remodelling involve different combinations of immune cells, chondrocytes, osteoblasts, osteoclasts, endothelial cells, and mechanical cues, making fracture repair a staged and potentially time-sensitive regenerative process. The stage-specific framework is summarised in **Figure 2**.

### Haematoma and inflammation

The earliest fracture haematoma is not passive extravasated blood but the initiating repair niche. Coagulation products, necrotic tissue, inflammatory cells, cytokines, and chemokines within the haematoma shape mesenchymal-cell recruitment, vascular ingrowth, and subsequent callus formation. Early fracture inflammation is not independent of time of day; it is likely shaped by local injury signals and systemic rhythmic inputs (78–81).

Macrophages and T cells may be particularly relevant in this phase. Early repair requires inflammatory initiation followed by resolution, reparative macrophage activity, angiogenesis, and progenitor recruitment. Immune-cell trafficking, cytokine release, and activation states are rhythmically regulated, and macrophage clocks influence inflammatory transcription and metabolism (29–34). T-cell subsets also participate in fracture inflammation, osteogenesis, and remodelling, although their temporal organisation within callus remains insufficiently mapped (41). Sleep loss, abnormal light, surgical stress, or endocrine disruption could therefore modify immune transitions, but this remains a fracture-specific hypothesis rather than an established outcome effect.

Direct time-resolved data on immune-cell profiles and clock-gene expression in human fracture haematoma remain scarce. Future studies should prioritise standardised sampling of human fracture haematoma or early callus tissue, combined with single-cell immune profiling, cytokine measurement, and clock-gene analysis, and should relate these data to early callus formation and inflammatory-resolution kinetics.

### Cartilaginous callus and endochondral ossification

Cartilaginous callus formation is the repair phase that most clearly reflects staged biological transition. Mesenchymal progenitors differentiate toward a chondrogenic lineage under hypoxic, inflammatory, and mechanically specific conditions, forming a proteoglycan- and type II collagen-rich soft callus. Chondrocytes then undergo hypertrophy, and the cartilaginous matrix is replaced by vascularised bone. Chondrocyte molecular clocks regulate cartilage homeostasis, matrix synthesis, and matrix degradation (35–37), providing a mechanistic entry point for considering temporal sensitivity in the soft-callus phase.

Extrapolation from articular cartilage or growth plate to fracture callus must be cautious. Articular cartilage is a relatively stable permanent tissue, and the growth plate is developmentally programmed. Fracture callus is a transient, injury-induced, inflammation-driven tissue shaped by mechanical environment. Existing cartilage rhythm evidence supports the hypothesis that the soft callus may be time-sensitive, but it does not prove that callus quality or endochondral ossification rate is determined by clock time. Future fracture-specific studies should examine BMAL1, PER2, REV-ERB, SOX9, COL2A1, ACAN, COL10A1, MMP13, and vascular-invasion markers at defined circadian times and relate them to callus mineralisation and mechanical strength.

### Hard-callus mineralisation

Hard callus formation marks the transition from a cartilaginous scaffold to mineralised bone. This phase involves hypertrophic chondrocyte clearance or transformation, vascular invasion, osteoprogenitor recruitment, osteoid deposition, and initial mineralisation. Angiogenesis is essential for bone regeneration, and endothelial function, vascular tone, permeability, and inflammatory adhesion are themselves under circadian regulation (38–40). The hard-callus phase should therefore be interpreted through the joint activity of osteoblasts, osteoclasts, endothelium, immune cells, and the mechanical environment.

Fixation stability, micromotion, loading, vascularisation, and immune transition jointly shape callus maturation. In intact mouse tibiae, loading during the active phase produced a modestly greater endocortical formation response than rest-phase loading and differentially regulated Sost and Dkk1, indicating time-of-day-dependent mechanoadaptation (71). This is relevant adjacent evidence, but it cannot be transferred directly to an unstable or incompletely bridged fracture. Fracture studies should therefore record fixation stability, loading dose and timing, vascularisation, callus mineralisation, and local clock readouts before testing phase-dependent mechanical or endocrine interventions.

### Remodelling and resorption rhythms

The remodelling phase converts woven callus into mechanically oriented lamellar bone and restores marrow and cortical architecture. Compared with earlier phases, it is closer to classical bone turnover: human CTX shows marked diurnal and intrinsic circadian variation, whereas P1NP and osteocalcin generally show smaller or less consistent rhythms (16–19). Coupled osteoclastic resorption and osteoblastic formation therefore make remodelling suitable for time-anchored measurement, but fracture remodelling remains distinct from steady-state turnover.

Fracture remodelling, however, is not identical to steady-state bone remodelling. Osteoclasts participate not only in pathological bone loss but also in cartilage callus clearance, woven bone reshaping, and marrow-cavity restoration. Suppressing bone resorption excessively during particular windows may increase apparent callus volume without improving structural maturity or mechanical quality. Melatonin studies illustrate this caution. Although melatonin has antioxidant, anti-inflammatory, and possible pro-osteogenic effects in some bone models, high-dose continuous melatonin impaired early mechanical recovery in a mouse femoral fracture model by suppressing RANKL-mediated callus remodelling (82, 83). This finding should not be simplified as “melatonin is harmful”; rather, it underscores phase dependence. During windows that require osteoclast-mediated callus shaping, excessive antiresorptive pressure may interfere with normal repair.

The remodelling phase therefore highlights a broader principle: the effects of a given intervention may depend on repair stage, dose, administration pattern, and callus maturity. Antiresorptive drugs, PTH analogues, melatonin, mechanical loading, and nutritional interventions should not be categorised simply as “bone-promoting” or “resorption-suppressing”. Future studies should combine longitudinal imaging, mechanical endpoints, bone turnover markers, and precise intervention timing to distinguish increased callus volume from improved structural maturation.

## Perioperative rhythm vulnerability and protection

**Figure 3:**
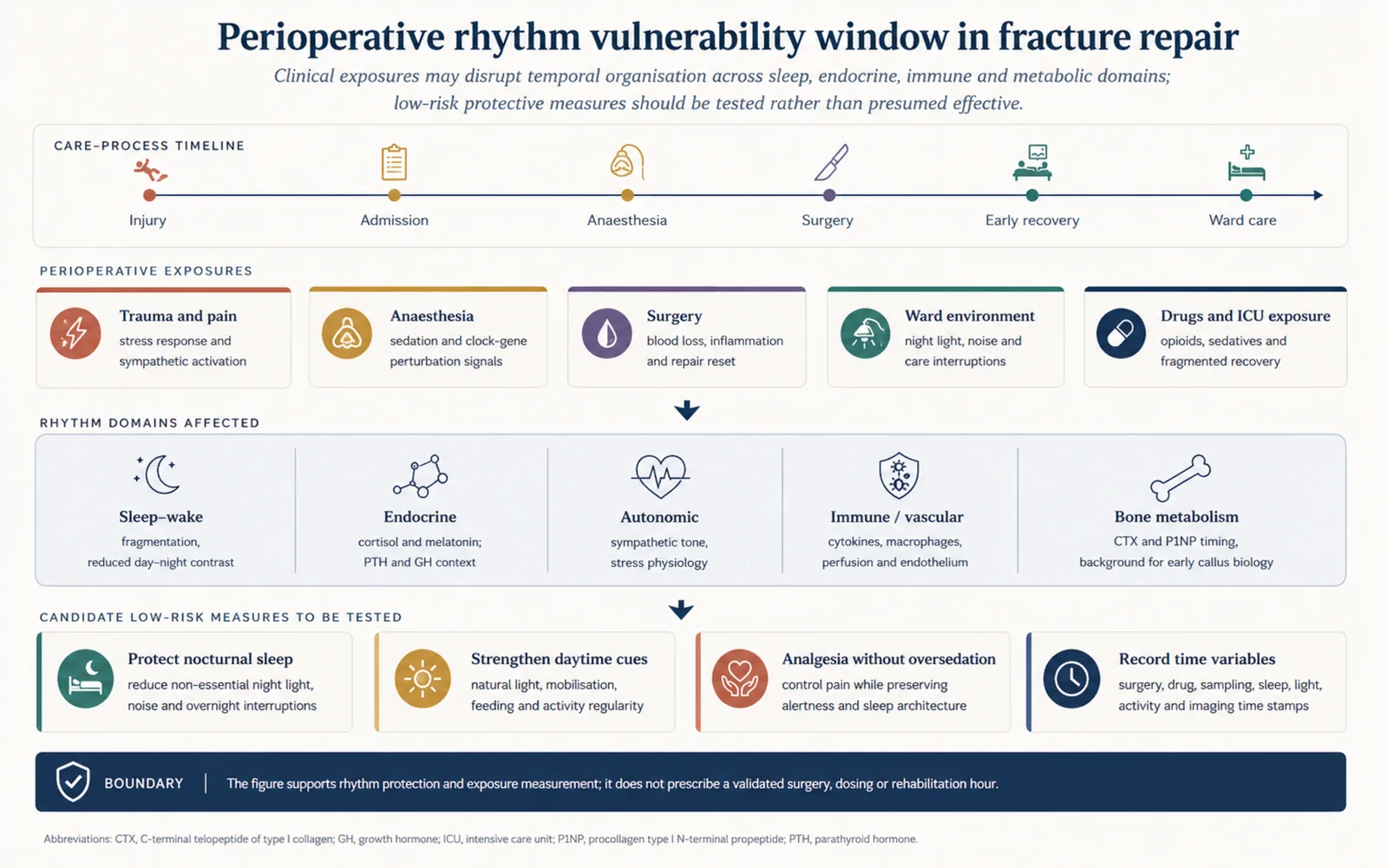
Perioperative rhythm vulnerability window in fracture repair. Trauma, anaesthesia, surgery, ward environment, medication exposure, sleep disruption, light exposure, and activity changes may affect sleep–wake, endocrine, autonomic, immune–vascular, and bone-metabolic domains. Candidate rhythm-protection measures should be tested prospectively against fracture-specific outcomes.

The perioperative period is the principal interface between fracture chronobiology and clinical care. Trauma, pain, emergency transfer, anaesthesia, surgery, blood loss, ward exposure, sleep disruption, and polypharmacy occur within a short interval and can perturb endocrine stress, immune function, activity, and sleep–wake organisation. This rhythm-vulnerable window provides a clinically realistic setting for measurement and low-risk protection studies. The perioperative vulnerability framework is summarised in **Figure 3**.

### Trauma, anaesthesia, and surgery

Trauma and surgery activate cortisol and sympathetic pathways, inflammatory mediators, and sleep disruption; cellular and animal studies also suggest that some anaesthetic agents can alter clock-gene expression or circadian outputs. Human perioperative evidence more consistently documents impaired sleep architecture and reduced day–night activity contrast (42–47). These studies rarely assess fracture healing, so anaesthesia timing and postoperative rhythm measures should be treated as exposures rather than presumed determinants of union.

### Hospital environmental disruption

Compared with the acute effects of surgery, rhythm disruption caused by the ward environment may be more sustained and less visible. Nocturnal light, noise, repeated vital-sign checks, pain, anxiety, opioids, sedatives, and intensive care exposure can fragment sleep and weaken the contrast between daytime activity and night-time rest. Older patients with hip fracture are especially vulnerable because they often have fragmented sleep, cognitive vulnerability, reduced melatonin secretion, multimorbidity, and postoperative delirium risk.

Melatonin and melatonin-receptor agonists have been studied in some perioperative and delirium-prevention contexts, but these studies generally do not assess fracture healing, callus formation, or nonunion as outcomes (48, 49). In fracture patients, it is therefore more appropriate to record sleep, light exposure, activity, and nocturnal interruptions as perioperative exposures than to frame melatonin as a bone-healing drug.

### Surgical timing versus circadian phase

The relationship between surgical time and fracture outcomes is easily overinterpreted. Many orthopaedic trauma studies compare daytime, night-time, or out-of-hours surgery from the perspective of care quality and perioperative safety, with mixed findings. In hip fracture, the more consistent evidence concerns time from admission to surgery and its association with mortality and complications, not the clock time at which surgery occurs (50–53). Timely surgery, team resources, injury severity, emergency status, and perioperative pathways may all influence outcomes. Apparent risks of night-time or out-of-hours surgery may reflect team fatigue, resource allocation, postoperative monitoring, or patient selection rather than endogenous circadian phase.

Accordingly, the benefit of early surgery should not be translated into a claim that a particular clock time is favourable for fracture healing. Studies of surgical timing and healing should distinguish emergency from elective procedures, care delay from circadian phase, and biological timing from system-level process variables. They should adjust for injury severity, anaesthetic technique, fixation method, team resources, intensive care exposure, and postoperative care, while using time-resolved endpoints such as early callus volume, mineralisation, bridging cortices, nonunion, and functional recovery.

### Rhythm protection before chronotherapy

In the absence of direct fracture chronotherapy evidence, the practical goal is to reduce avoidable disruption: limit non-essential nocturnal light, noise, and care interruptions; provide analgesia without excessive sedation; support daytime light and mobilisation; protect sleep continuity; and record medication, sampling, activity, and imaging times. These measures are clinically feasible but still require prospective evaluation against fracture-specific outcomes.

## Time-anchored translational opportunities

Time-anchored strategies are not equivalent to mature chronotherapy. We therefore distinguish human fracture-specific evidence without established circadian timing, direct preclinical fracture-timing evidence, indirect skeletal or physiological evidence, and hypothesis-generating proposals. This distinction identifies variables suitable for prospective studies without converting adjacent evidence into clinical schedules. The candidate time-anchored strategies are summarised in **Table 2**.

**Table 2:** Potential time-anchored strategies, current evidence basis, and near-term research priority.

Strategy or exposure	Time anchor to record	Biological or clinical rationale	Current evidence basis	Near-term research priority
Antiresorptive drugs	Initiation after fixation; study-relevant dose times; BTM sampling	Early initiation generally does not delay union (54–56)	Human fracture-specific initiation evidence; daily circadian timing untested	Assess remodelling quality and function, not only nonunion
PTH analogues	Start date; injection and sampling times; repair stage	Fracture-site clock responsiveness (15); mixed fracture trials (57–59); osteoporosis timing studies (63, 64)	Human fracture efficacy evidence without circadian comparison; indirect timing evidence	Prospective timing capture before a randomised timing trial
NSAIDs and analgesia	First and key doses; duration; repair stage; sleep	Pain control may support sleep; inflammatory suppression may be stage-dependent	Direct murine active/rest-phase fracture study (62); mixed broader clinical evidence (60)	Replicate across drugs/models; do not convert mouse phase to human clock time
Sleep and light protection	Night light/noise; interruptions; daytime light/activity	May preserve sleep–wake and endocrine–immune organisation (42–49)	Indirect but feasible; fracture-healing benefit unproven	Pragmatic feasibility trials with fracture-specific exploratory outcomes
Vitamin D and nutritional adequacy	Baseline status; support start; dosing and sampling time	Corrects deficiency and supports the repair environment (61)	General bone-health evidence; not chronotherapy	Record deficiency and support consistently in fracture cohorts
Movement, loading, and feeding	Loading eligibility; activity phase; dose, rate, duration; meal relation	Intact-bone and postmenopausal studies show temporal/mechanical/nutritional modulation (63, 64, 71–77)	Indirect; no validated post-fracture schedule	Wearable and rehabilitation-log studies under fixation-specific safety constraints
Vulnerable populations	Chronotype; sleep/light/activity; study-relevant drug and sampling times	Ageing, diabetes, osteoporosis, and menopause may reduce repair reserve (65–70)	Plausible effect modification; fracture-specific rhythm data sparse	Prioritise prospective time-anchored cohorts
Surgical and care-process timing	Injury, admission, anaesthesia, surgery, fixation, ICU and discharge	Separates delay and system effects from biological phase (50–53)	Human process evidence; substantial confounding	Prospective adjusted studies using callus and functional endpoints

BTM, bone turnover marker; CTX, C-terminal telopeptide of type I collagen; NSAIDs, non-steroidal anti-inflammatory drugs; P1NP, procollagen type I N-terminal propeptide; PTH, parathyroid hormone.

### Antiresorptive initiation versus daily timing

Whether bisphosphonates impair fracture healing has long been clinically debated. Randomised trials and meta-analyses generally suggest that early bisphosphonate initiation after fracture surgery does not substantially delay radiographic or clinical healing and does not clearly increase delayed union or nonunion. Reviews of osteoporosis therapy after fracture similarly suggest that most human studies do not show clear adverse effects of early bisphosphonate use on acute fracture healing (54–56). This evidence helps mitigate concern that early antiresorptive treatment necessarily impedes union.

However, these studies address whether bisphosphonates can be initiated within weeks after surgery; they do not address whether the daily administration time affects fracture healing. Evidence on antiresorptive timing should therefore inform initiation windows, not be extrapolated to circadian dosing recommendations. Future studies should record initiation date, administration time, biomarker sampling time, and imaging outcomes to determine whether antiresorptives affect callus remodelling quality during specific repair phases.

### PTH analogues

PTH analogues, particularly teriparatide, are plausible candidates for timing studies. Human fracture trials have suggested earlier callus formation or shorter radiographic healing in selected settings, but systematic reviews remain inconsistent because fracture type, fixation, treatment start, endpoint definitions, and sample size vary (57–59). In postmenopausal osteoporosis, small studies found that morning versus evening teriparatide altered 24-h bone-turnover profiles, and a 12-month trial reported a larger lumbar-spine BMD gain with morning dosing (63, 64). These outcomes are not fracture healing.

Available evidence does not establish that morning versus evening PTH administration improves callus formation, mechanical strength, union time, or functional recovery. PTH should therefore be treated as a mechanism-supported candidate for prospective timing studies. Standardised fracture cohorts can record initiation date, injection time, internal or behavioural phase indicators, biomarker sampling, and callus trajectory before considering a randomised timing comparison.

### NSAIDs and the inflammatory window

The relationship between non-steroidal anti-inflammatory drugs (NSAIDs) and fracture healing depends on drug, dose, duration, model, fracture type, and patient risk (60). A murine tibial fracture study directly compared active- and rest-phase NSAID administration and reported better pain, callus, and mechanical outcomes when dosing was confined to the active phase, whereas rest-phase dosing impaired healing (62). This is unusually direct timing evidence, but it is a single preclinical model and active/rest phase in nocturnal mice cannot be translated into human morning/evening dosing.

Adequate analgesia can reduce stress and support sleep and mobilisation, whereas prolonged suppression of early inflammatory signalling may impair repair. Prospective studies should record first dose and prespecified key doses, duration, cumulative exposure, repair stage, sleep state, and callus endpoints. The immediate implication is improved exposure measurement, not a fixed NSAID dosing hour.

### Vitamin D and nutritional adequacy

Vitamin D deficiency is associated with abnormal bone metabolism, fracture risk, and falls, and correction is clinically reasonable in deficient patients. Evidence for accelerated radiographic healing or improved callus quality remains inconsistent (61). Vitamin D, calcium, energy, and protein adequacy should therefore be treated as components of the repair environment; studies should record baseline status, supplementation or nutritional-support start, dosing pattern, and sampling time rather than infer a chronotherapeutic effect.

### Movement–feeding–endocrine coupling

Mechanical loading is central to bone repair, but temporal evidence comes mainly from intact-bone or osteoporosis research. In mice, active-phase tibial loading produced a modestly greater site-specific endocortical response than rest-phase loading (71). In postmenopausal women, higher walking intensity was associated with more favourable preservation or accrual of bone mineral density (74). In a separate acute study, a morning, post-meal, longer-duration walking bout with sufficient momentum produced a more anabolic bone-turnover marker profile, including under downhill-loading conditions (72). Neither human study involved acute fracture rehabilitation. Together, these findings identify testable dimensions—phase, loading intensity, rate, duration, direction, and recovery interval—but do not establish a rehabilitation schedule after fracture.

Feeding can also modify skeletal readouts. Post-meal exercise increased an osteogenic marker ratio in diabetic postmenopausal women, and refeeding potentiated load-induced cortical formation in young mice, although the effect was not reproduced in aged mice (73, 75). Meals acutely suppress CTX, and GLP-2 can reduce nocturnal resorption markers in postmenopausal women (76, 77). These responses may reflect event-related or ultradian endocrine dynamics rather than circadian phase; the temporal scales should not be treated as interchangeable.

For fracture rehabilitation, movement remains stage-, fixation-, and safety-dependent. Protocols derived from uninjured bone or osteoporosis studies cannot be transferred directly to patients with unstable fractures or recent fixation; loading rate, direction, duration, feeding relation, and clock time require fracture-specific safety and efficacy testing. The near-term question is whether, after stability and loading eligibility are established, prospectively measured activity phase, loading dose, feeding relation, and PTH state explain variation in callus and function. Wearables and rehabilitation logs can capture these exposures without prescribing an unvalidated regimen.

### Vulnerable populations

Older adults, patients with diabetes, postmenopausal women, and patients with osteoporosis are priority populations for time-anchored fracture research. They are not yet proven to benefit from rhythm intervention. Rather, they have higher fracture risk, greater risk of impaired repair, and a heavier burden of rhythm disruption. Osteoporosis, diabetic skeletal fragility, skeletal ageing, and postmenopausal bone turnover may reduce repair reserve and amplify the impact of perioperative rhythm disruption (65–69). Epidemiological associations between shift work or long-term rhythm disruption and low bone mineral density further suggest that chronic rhythmic state may influence bone health (70).

The emphasis should therefore be on prospective observation and low-risk rhythm-protection studies, not on claiming that vulnerable patients require chronotherapy. Cohorts of older patients with hip fracture, patients with diabetic lower-limb fractures, and postmenopausal women with osteoporotic fractures could systematically record sleep, light, activity, drug timing, blood sampling, bone turnover markers, and imaging endpoints. If these variables consistently explain differences in callus formation, mineralisation, or functional recovery, targeted intervention trials can be justified.

## Discussion

**Figure 4:**
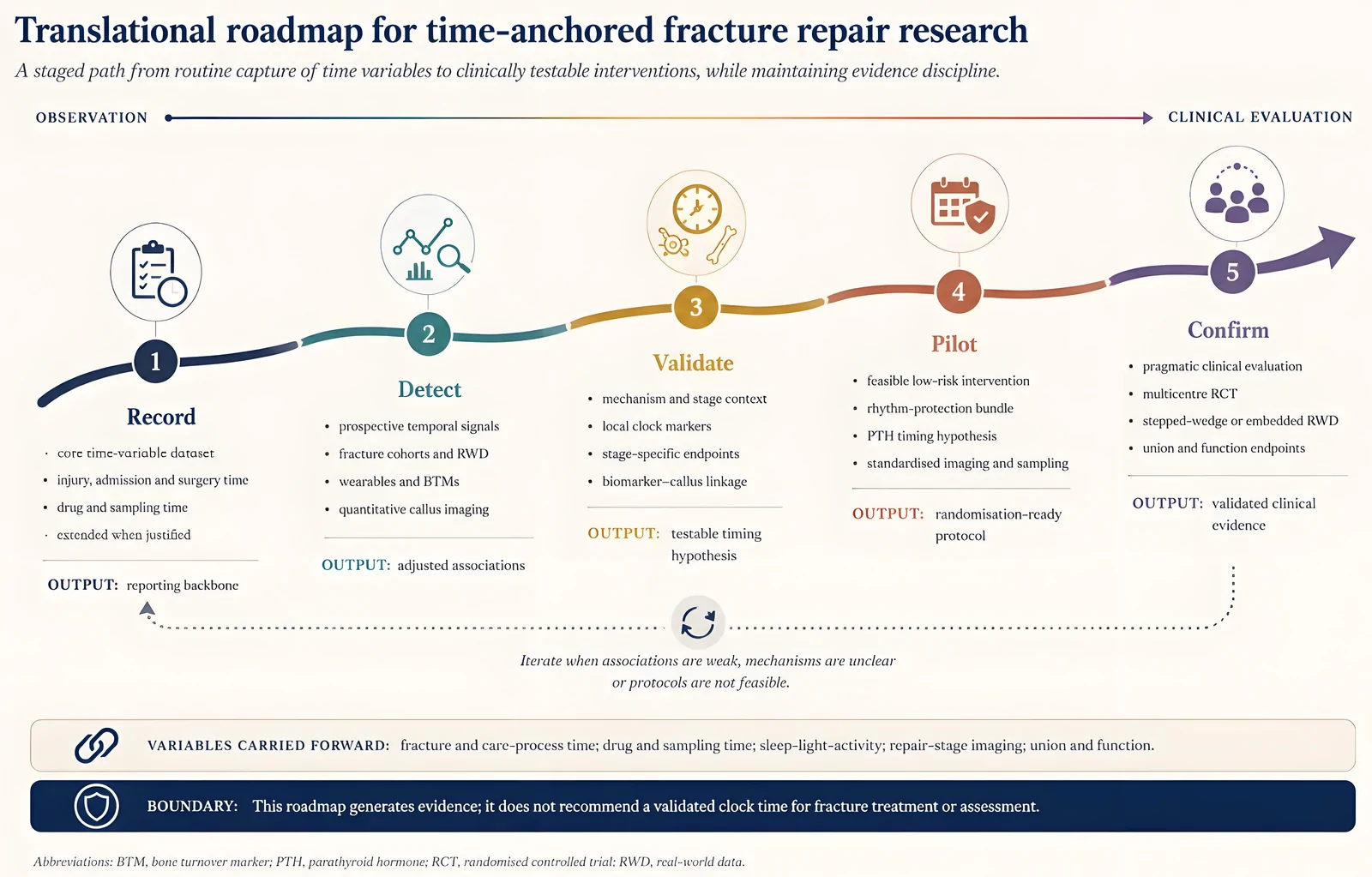
Translational roadmap for time-anchored fracture repair research. “Record” denotes standardised capture of core time variables, whereas “Detect” denotes prospective analysis of adjusted temporal signals; standardised reporting applies across all stages. Signals then require mechanistic or cross-cohort validation before low-risk pilot and confirmatory evaluation. RCT, randomised controlled trial; RWD, real-world data. The roadmap generates evidence and does not recommend a treatment or assessment hour.

The principal translational gap is not the absence of a single optimal treatment hour, but the routine omission of time as a measurable covariate. A practical roadmap begins with a core dataset that most fracture studies can collect, adds extended rhythm measures only when justified, and progresses from adjusted associations to mechanistic validation and low-risk trials. The proposed translational roadmap is shown in **Figure 4**.

### From time-agnostic to time-anchored fracture research

Future studies should distinguish three related but non-interchangeable dimensions. Circadian-related timing includes external clock time and behavioural or zeitgeber timing (sleep, light, meals, and activity) as well as internal phase measured by markers such as dim-light melatonin onset; clock time alone is not a biological-phase measure. Care-process time includes injury, admission, anaesthesia, surgery, medication, loading, rehabilitation, and discharge. Repair-stage time includes inflammation, cartilaginous callus, mineralisation, and remodelling.

This distinction is critical when interpreting existing evidence. “Early surgery” in hip fracture primarily addresses admission-to-surgery delay, not circadian phase. Early bisphosphonate initiation after surgery addresses the treatment initiation window, not the daily administration time. Differences between morning and afternoon CTX sampling are biomarker timing issues. Without separating these dimensions, apparent “time effects” may reflect care pathways, disease severity, or sampling bias rather than biological rhythmicity.

We therefore propose time-anchored research as a near-term goal. This does not mean that every fracture study must become a complex circadian experiment. Rather, animal studies, clinical cohorts, randomised trials, and real-world studies should record key time variables and incorporate them into adjustment, stratification, or sensitivity analyses. This approach avoids overinterpretation while establishing a reproducible basis for future time-based interventions.

### Core and extended reporting framework

The core clinical dataset should remain feasible: injury and major care times; the first dose and prespecified key administration times for study-relevant exposures; biological sampling time and fasting status; and actual intervals from injury or fixation to imaging, mobilisation, loading, and outcome assessment. Sleep, light, chronotype, wearables, detailed feeding, endocrine phase markers, and advanced rhythm modelling belong to an extended dataset for prospective chronobiology studies. The reporting framework is summarised in **Table 3**.

**Table 3:** Core and extended time-variable reporting framework for fracture studies.

Dataset tier	Variables to record	Recommended reporting	Why it matters
Core: injury and care pathway	Injury, admission, anaesthesia, surgery, fixation completion	Exact date and 24-h time; emergency/elective status; admission-to-surgery interval	Separates care-process time from clock time
Core: study-relevant treatment and sampling	First dose and prespecified key doses; biological samples	Date/time, dose, route; sampling time, fasting, posture, recent sleep/food	Prevents exposure and biomarker timing misclassification
Core: repair-stage and outcomes	Fracture/fixation; mobilisation/loading start; imaging; union and function	Actual intervals from injury and fixation to each exposure and assessment	Aligns measurements with biological repair stage
Core: preclinical studies	Light–dark cycle or ZT; fracture induction; intervention; harvest/imaging; sex and age	Report clock time and ZT/active-rest phase without pooling across phases	Supports replication and cross-study interpretation
Extended: patient rhythm state	Chronotype; sleep–wake timing; fragmentation; naps	Validated questionnaire, diary, or actigraphy	Characterises behavioural timing and rhythm disruption
Extended: environment	Light, noise, interruptions, ICU exposure, sedatives, ward transfer	Logs or objective monitoring when relevant	Captures modifiable inpatient zeitgebers
Extended: activity, loading, and feeding	Steps, loading dose/rate, rehabilitation sessions, meal timing	Wearable plus rehabilitation and meal logs	Tests movement–feeding–endocrine hypotheses
Extended: phase and analysis	Melatonin/cortisol phase; repeated biomarkers; prespecified time models	Use rhythm, mixed, time-dependent, or competing-risk methods only when supported	Prevents overengineering while enabling specialised chronobiology studies

ICU, intensive care unit; ZT, zeitgeber time. Core variables are intended for routine reporting in most clinical or preclinical fracture studies, whereas extended variables are recommended for specialised prospective chronobiology studies when justified by the research question.

Sampling time should be fixed for CTX, P1NP, osteocalcin, cortisol, melatonin, and selected inflammatory mediators whenever feasible. Otherwise, exact time, fasting status, posture, recent sleep, and food intake should be recorded and addressed analytically. Imaging should be anchored to the actual interval from injury, fixation, and loading rather than reported only as a nominal postoperative week.

The preclinical core comprises the light–dark cycle or zeitgeber reference, fracture induction, dosing or intervention, tissue harvest or imaging time, sex, and age. Samples collected at different biological phases should not be pooled without explicit justification. Zeitgeber time, active/rest phase, and human clock time must be reported separately and should not be translated mechanically across nocturnal rodents and humans.

### Near-term translational priorities

Near-term studies should prioritise feasible questions embedded within standard care and use fracture-specific endpoints.

First, medication-timing studies should separate initiation window from daily phase. Existing human evidence supports the safety question of early antiresorptive initiation, while the murine NSAID study provides direct but unreplicated active/rest-phase evidence (54–56, 62). Standardised cohorts can record key dosing and biomarker times before randomising a timing schedule; callus mineralisation, mechanical or functional recovery, and adverse events should accompany union.

Second, a perioperative sleep–light protection feasibility trial is suitable for older fragility-fracture patients. A pragmatic bundle could reduce non-essential night interruptions, strengthen daytime light and activity cues, and avoid oversedation. Feasibility and sleep–activity contrast should be primary early outcomes, with inflammatory markers, time-standardised bone markers, and early callus or functional measures as exploratory endpoints.

Third, prospective surgical-time studies should separate clock time from admission-to-surgery delay and adjust for emergency status, injury severity, anaesthesia, fixation, team resources, intensive-care exposure, and postoperative pathway. Their value is to test whether an adjusted temporal signal persists for callus or function, not to recommend daytime scheduling from unadjusted out-of-hours comparisons.

Fourth, movement–feeding–endocrine studies should begin only after fixation stability and loading eligibility are defined. Wearables and rehabilitation logs can quantify activity phase, loading dose, meal relation, sleep, and PTH treatment time. Fracture-specific imaging and functional endpoints can then determine whether signals observed in intact bone or osteoporosis cohorts warrant controlled timing trials.

The purpose of these questions is not to create immediate clinical prescriptions, but to determine whether time variables explain part of the heterogeneity in fracture repair. Stable associations with callus formation, inflammatory resolution, mineralisation, or function would justify randomised timing interventions. Conversely, unstable or process-driven associations would be equally informative by preventing misinterpretation of complex care pathways as biological circadian effects.

### Measurement and analysis

Implementation can be proportionate. Core studies need accurate time stamps and time-resolved fracture outcomes; specialised studies may add chronotype questionnaires, actigraphy, light monitoring, endocrine phase markers, and quantitative callus imaging (84–88).

Biomarkers should be sampled at fixed times when possible or adjusted for collection time, fasting, sleep, and food intake. Wearables can estimate sleep continuity, day–night activity contrast, phase, and fragmentation, but these measures should be prespecified rather than collected indiscriminately (89–91).

Analysis should match the design. Mixed-effects models suit repeated biomarkers or imaging; cosinor or related methods require genuinely periodic sampling; and time-varying exposures may require time-dependent models. Causal diagrams should guide confounder selection, with advanced causal or competing-risk methods reserved for questions and datasets that support them (92–98). Future observational, real-world, or scoping work should follow the relevant reporting standard rather than treating this narrative framework as a substitute (99–101).

Three interpretive errors should be avoided. Care-process time is not internal circadian phase; rodent zeitgeber or active/rest phase is not equivalent to a human morning/evening prescription; and acute meal- or exercise-related endocrine responses may be ultradian or event-driven rather than circadian. Indirect physiology should therefore generate fracture-specific hypotheses, not treatment times.

## Limitations

This review has several limitations. First, it is a narrative review rather than a systematic review. Although we used the SANRA framework to improve the transparency of evidence searching and synthesis, we did not conduct formal risk-of-bias assessment, evidence grading, or quantitative meta-analysis. We therefore cannot estimate the pooled effect of any intervention or recommend a specific clock time for surgery, drug administration, sampling, or rehabilitation.

Second, direct evidence in fracture chronobiology remains limited and relies heavily on one ex vivo mouse Per2::Luc fracture model. Time-resolved human data on local clocks, immune profiles, callus transition, vascularisation, remodelling, and healing outcomes are scarce. Much of the supporting evidence derives from bone homeostasis, osteoporosis, intact-bone loading, cartilage, immune or vascular biology, sleep physiology, and perioperative research; these sources can support hypotheses but not fracture treatment schedules.

Third, time variables are deeply intertwined with clinical workflow. Injury time, admission time, surgical clock time, surgical delay, emergency status, anaesthesia resources, team availability, intensive care exposure, postoperative care pathway, and baseline severity are closely linked. Observational studies that do not record and adjust for these variables may misclassify care-system effects, selection bias, or sampling bias as biological rhythm effects.

Fourth, fracture type, fixation method, repair mode, age, metabolic status, nutrition, and rehabilitation pathway vary substantially. The four-stage callus framework primarily reflects secondary fracture healing and should not be applied uncritically to primary cortical or predominantly intramembranous repair. In addition, nocturnal rodent active/rest phases, human clock time, behavioural timing, and internal circadian phase are not interchangeable.

Fifth, the perioperative rhythm-protection and time-anchored reporting framework proposed here is a research and management framework, not a validated therapy. Reducing nocturnal light and noise, protecting sleep, and recording dosing and sampling time are biologically plausible and feasible, but whether they improve callus quality, nonunion risk, or long-term function remains to be tested prospectively. These limitations do not weaken the translational value of chronobiology; rather, they underscore the need to avoid prematurely converting attractive mechanisms into clinical prescriptions.

## Conclusions

Fracture repair is temporally organised, and its endocrine, immune, vascular, and skeletal components show rhythmic regulation to varying degrees. Per2::Luc oscillation and PTH-responsive phase modulation provide direct preclinical evidence that a local clock signal can operate in the mouse fracture niche, while human bone-marker physiology establishes the importance of timed measurement. Neither evidence stream demonstrates an optimal treatment hour.

Current evidence, however, is insufficient to support mature clinical recommendations for fracture chronotherapy. No specific clock time can currently be inferred for surgery, drug administration, blood sampling, loading, or rehabilitation to improve fracture healing. The more defensible and translationally useful direction is to move fracture research and perioperative care from time-agnostic to time-anchored practice. Clinically, avoidable perioperative rhythm disruption should be reduced and sleep-wake organisation protected. Scientifically, injury, admission, anaesthesia, surgery, drug administration, sampling, sleep, light, activity, loading, and imaging times should be recorded systematically.

The near-term goal of fracture chronobiology is therefore not to prescribe unvalidated chronotherapy, but to build a measurable, adjustable, and reproducible time-anchored research framework. Only after key time variables are recorded and reliably linked to callus formation, mineralisation, remodelling, nonunion, and functional recovery can fracture chronobiology progress from mechanistic hypothesis to testable clinical translation. Time-anchored fracture research is the necessary intermediate step between mechanistic chronobiology and responsible clinical chronotherapy.
